# Survival benefit of secondary prevention medical therapy in takotsubo cardiomyopathy: a Bayesian network meta-analysis

**DOI:** 10.1093/ehjopen/oeaf040

**Published:** 2025-04-16

**Authors:** Daud Mutahar, Ammar Zaka, Stephen Bacchi, Brandon Stretton, Joshua G Kovoor, Aashray K Gupta, Naim Mridha

**Affiliations:** Faculty of Health Sciences and Medicine, Bond University, 14 University Drive, Robina, QLD 4216, Australia; Department of Cardiology, Gold Coast University Hospital, 1 Hospital Boulevard, Southport, QLD 4215, Australia; Department of Cardiology, Gold Coast University Hospital, 1 Hospital Boulevard, Southport, QLD 4215, Australia; Massachusetts General Hospital, 55 Fruit St, Boston, MA 02114, USA; Royal Adelaide Hospital, Port Rd, Adelaide, SA 5000, Australia; Department of Cardiology, Westmead Hospital, Cnr Hawkesbury Road and Darcy Road, Westmead, NSW 2145, Australia; Royal North Shore Hospital, Reserve Rd, St Leonards, NSW 2065, Australia; Department of Cardiology, Gold Coast University Hospital, 1 Hospital Boulevard, Southport, QLD 4215, Australia; Department of Cardiology, The Prince Charles Hospital, 627 Rode Rd, Chermside, QLD 4032, Australia

**Keywords:** Takotsubo cardiomyopathy, Heart failure, Pharmacotherapy, Acute coronary syndrome

## Abstract

**Aims:**

Takotsubo cardiomyopathy (TTC) is a form of transient left ventricular systolic dysfunction without evidence of complicated coronary artery disease. Efficacy of medical therapy in secondary prevention of all-cause mortality is not well established. We performed a systematic review and network meta-analysis to compare survival benefit of secondary prevention medical therapy in patients with TTC.

**Methods and results:**

PubMed, Embase, and Cochrane were searched up to 6 January 2024. Eligible studies included multivariable-adjusted or propensity-matched studies of patients receiving medical therapy with beta-blockers, angiotensin-converting enzyme inhibitors (ACE) or angiotensin receptor blockers (ARBs), aspirin, and statins after an index presentation with TTC. The primary outcome was all-cause mortality at any time point. Secondary outcome was TTC recurrence. Random-effect hierarchical Bayesian meta-analysis was performed. We identified 13 observational studies. Takotsubo cardiomyopathy mortality was reported in 435 (4.7%) out of 9237 patients, across a median follow-up of 2.18 years. Mean age was 69.7 ± 12.5 years, and 7906 patients (90.7%) were females. Beta-blockers were associated with a statistically significant reduction in mortality compared to control [hazard ratio (HR) 0.65, 95% confidence interval (CI) (0.55–0.77)]. ACE inhibitors/ARBs showed a nonsignificant trend towards mortality reduction [HR 0.76, 95% CI (0.54–1.07)]. Statins [HR 0.96, 95% CI (0.77–1.19)] and aspirin [HR 0.87, 95% CI (0.55–1.38)] showed no significant mortality benefit. Bayesian probability ranks favoured beta-blockers as the most effective treatment for TTC mortality prevention.

**Conclusion:**

This review highlights the modest efficacy of secondary prevention medications in the management of TTC, as ACE or ARBs, beta-blockers, aspirin, and statins failed to demonstrate comparative mortality benefit. Randomized controlled trials are needed to confirm efficacy of pharmacotherapy in this vulnerable patient cohort.

## Graphical Abstract

**Graphical Abstract:**
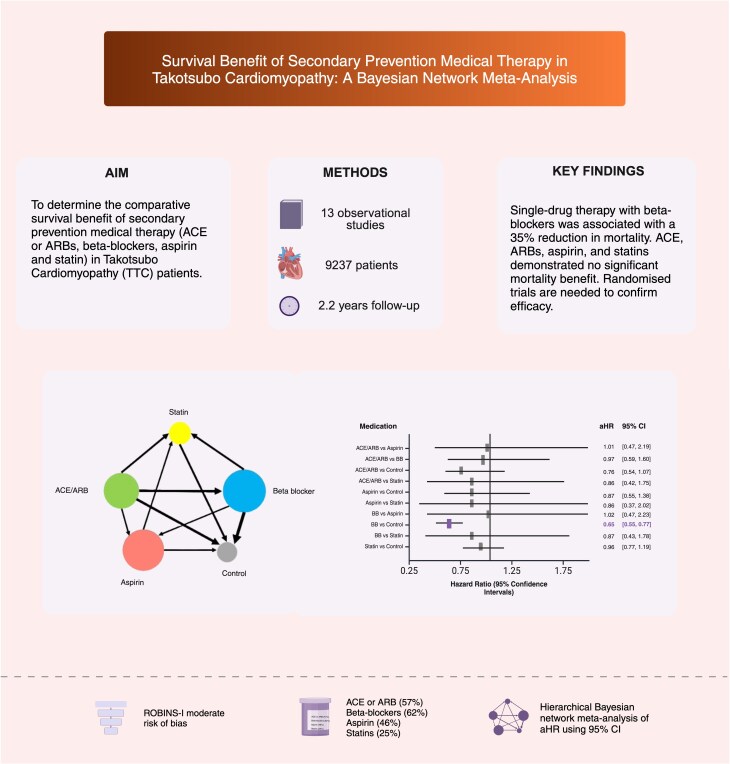

## Introduction

Takotsubo cardiomyopathy (TTC), also known as stress cardiomyopathy, is a heterogeneous acute cardiac syndrome characterized by transient left ventricular systolic dysfunction.^1–4^ Takotsubo cardiomyopathy often presents similarly to acute coronary syndrome (ACS) yet occurs in the absence of obstructive coronary artery disease (CAD) or acute plaque rupture.^5–7^ Historically believed be a benign disease, studies have shown that TTC has morbidity and mortality rates that are comparable to those of ACS, based on inconsistent data.^8–13^ Higher mortality rates are often observed in secondary forms of TTC, which are typically precipitated by acute medical stressors such as sepsis or surgery.^14,15^

Based on conflicting observational data and early mechanistic studies, contemporary guidelines recommend careful initiation of pharmacotherapy where significant predictors of ‘severe TTC’ are present; left ventricular ejection fraction (LVEF) of <35%, significant LVOTO gradients (>40 mmHg), suspected bystander coronary artery disease, normal-high systemic vascular resistance and complicating tachyarrhythmia.^11^ Angiotensin-converting enzyme inhibitors (ACE) or angiotensin receptor blockers (ARBs) have been associated with improved survival at 1-year after propensity matching, while beta-blockers have demonstrated inconsistent results.^12^ In a large observational study based on the InterTAK registry, one-third of patients on post-discharge beta-blockers experienced TTC recurrence.^12^ ACE or ARBs have been associated with inconsistent TTC recurrence rates, and a recent meta-analysis found no benefit for combination ACE or ARB and beta-blocker therapy in reduction of TTC recurrence.^16^ Furthermore, the mortality benefit for aspirin and statins is also uncertain,^17^ and currently these medications are only recommended if concomitant severe coronary artery disease is also present.^11^

To date, no randomized controlled trials have been conducted to establish the optimal management strategy for patients with TTC,^11^ and current guidelines are based on an inconsistent sample of observational data. We therefore performed a systematic review and network meta-analysis to consolidate the existing evidence, and to determine the relative survival benefit of secondary prevention medical therapy (ACE or ARBs, beta-blockers, aspirin, and statin) in TTC patients.

## Methods

This systematic review and meta-analysis is reported according to the Preferred Reporting Items for Systematic Reviews and Meta-Analyses (PRISMA) and Meta-analysis Of Observational Studies in Epidemiology (MOOSE) statement guidelines.^18,19^ This review is registered with PROSPERO (CRD42024600925) and did not require institutional board review.

### Search strategy

A comprehensive search strategy was designed and conducted using PubMed, Embase, Web of Science, and Cochrane databases from inception to 6 January 2024. We manually searched the references cited in the previous reviews and other important studies related to this subject. We did not need to contact the corresponding authors of the studies, as the relevant information was easily accessible from the original studies. We trialled the following search terms in varying combinations:

(‘Takotsubo Cardiomyopathy’ OR ‘Takotsubo Syndrome’ OR ‘TTS’ OR ‘apical ballooning syndrome’ OR ‘broken heart syndrome’ OR ‘stress cardiomyopathy’) AND (‘Prognosis’ OR ‘Recurrence’ OR ‘Mortality’ OR ‘Survival’ OR ‘Pharm*’).

The detailed search strategy, search terms used, and inclusion and exclusion criteria are provided in the supplementary materials.

### Study eligibility

We included all multivariable-adjusted or propensity-matched studies with patients receiving medical therapy with ACE or ARB, beta-blockers (BB), aspirin, and statins as discharge pharmacotherapy after an index presentation with TTC, and provided risk estimates or incidence for all-cause mortality. Studies were included if they enrolled patients consistent with but not limited to the Takotsubo Italian Network (TIN) register, Heart Failure Association, European Society of Cardiology, or InterTAK TTC diagnostic criteria.^5^ We also excluded review articles, abstracts without full text available, and studies not in English. No minimum follow-up time was specified.

### Outcomes

The primary outcome was all-cause mortality at any time point. Outcome definitions according to original studies are detailed in supplementary materials.

### Data extraction and quality assessment

Two reviewers (A.Z. and D.M.) screened all the titles and abstracts independently. This was performed with a free-to-use web application (Rayyan, Qatar Computing Research Institute, Ar-Rayyan, Qatar). Conflicts were resolved by inclusion of a third reviewer (N.M.). This process was followed by the full text review of the selected articles by the two independent reviewers (A.Z. and D.M.). We then extracted the data from selected studies using a standardized, pilot-tested extraction template. Data were extracted by two independent reviewers (A.Z. and D.M.), and conflicts were resolved through discussion or a third reviewer (N.M.). Reviewers extracted the data with regard to inclusion criteria, total number of patients, duration of follow-up, pharmacological treatment at the moment of discharge, during follow-up, and at the time of TTC all-cause mortality and TTC recurrence. We did not need to contact authors for missing data. Two reviewers (D.M. and A.Z.) assessed quality of included studies by using the ROBINS-I tool.^20^ Disagreements between reviewers for classifications were resolved by consensus.

### Statistical analysis

A hierarchical Bayesian network meta-analysis was conducted using R, utilizing a random effects model due to heterogeneity in population sample size and baseline demographics. Bayesian network was utilized over a frequentist model to synthesize evidence from heterogeneous observational studies, as it allows for the integration of prior information and the generation of posterior probabilities, which enhance the assessment of relative treatment effects.^21^ This method facilitates the estimation of both direct and indirect comparisons, which is particularly relevant for TTC where there is no direct RCT comparison of treatments.^11^ Additionally, Bayesian approaches offer probabilistic treatment rankings that improve interpretability, compared to frequentist methods, which can be more sensitive to sample size constraints and less effective in managing complex correlation structures.^22^ Mortality rates and multivariate-adjusted hazard ratios were extracted for both case (TTC patients receiving medical therapies) and control (TTC patients not receiving medication). Results are reported as hazard ratios (HRs) with 95% confidence intervals (CIs) using random effects model for direct comparisons among ACE or ARB, beta-blockers, aspirin, and statins, and adjusted hazard ratios were pooled for comparison with control, with statistical significance set at *P* < 0.05. The network graph was designed with node sizes proportional to the number of participants in each intervention and line thickness proportional to the number of studies comparing each treatment arm. Forest plots for the analysed outcomes and the network graph were obtained, illustrating HRs and 95% CIs for the direct comparisons among the five interventions (including control). Heterogeneity among studies was assessed using the *I*² statistic. A Surface Under the Cumulative Ranking (SUCRA) table was generated to rank treatments based on their effectiveness in the secondary prevention of TTC. A SUCRA of 90% means that the treatment of interest achieves 90% of effectiveness or safety relative to other interventions. Thus, the larger the SUCRA value, the higher the rank of the treatment, indicating a safer or more effective treatment.

## Results

### Search results

**Figure 1:**
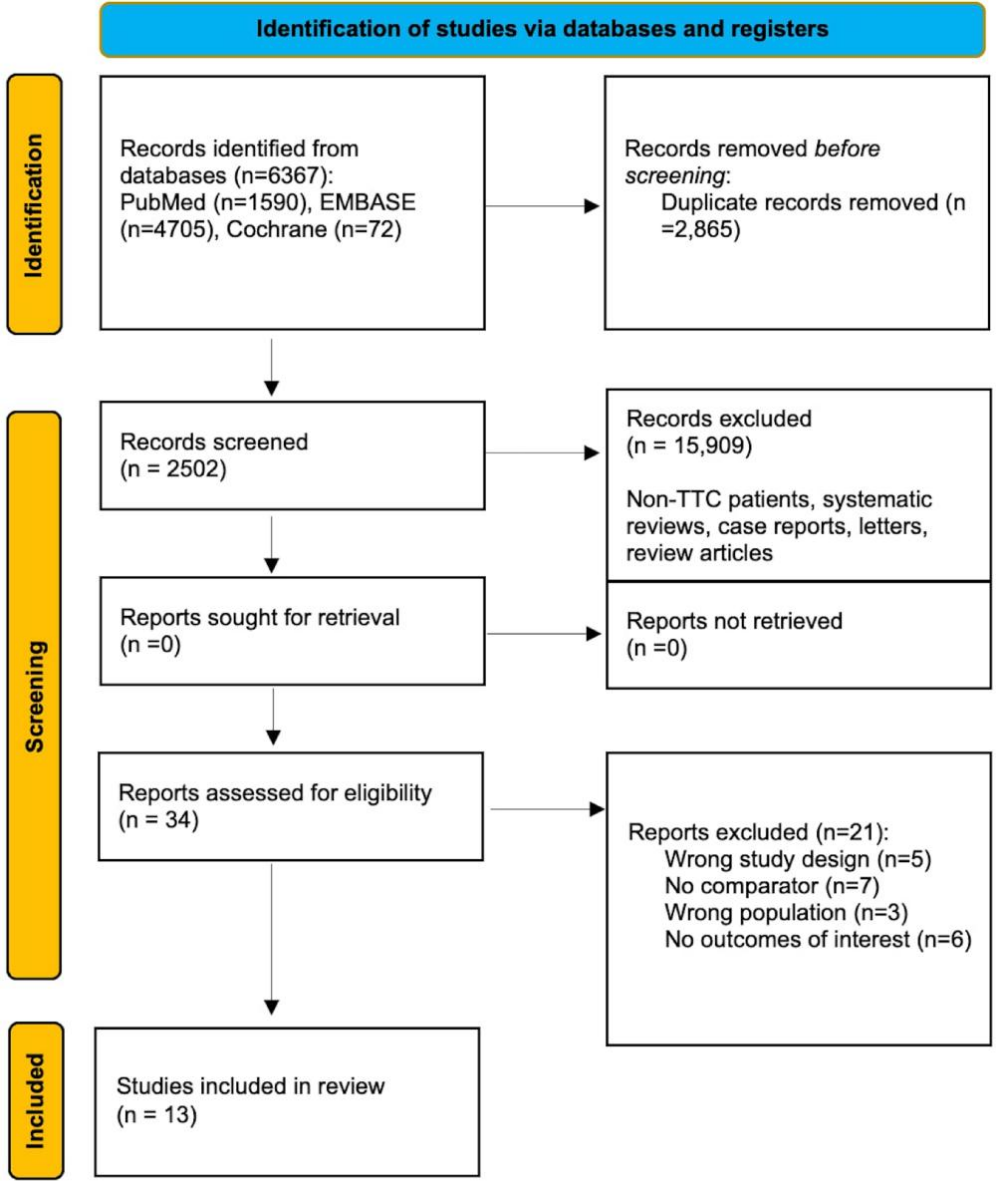
Study assessment and inclusion flowchart.

The literature search yielded 2502 unique studies after removal of duplicates, and 13 studies were included in this systematic review and meta-analysis (see *Figure 1*). Detailed rationale for exclusion of full-text studies is provided in the Supplementary material.

### Characteristics of included studies

A total of 13 observational studies were included in this systematic review,^17,23–34^ including 9237 patients. The weighted median follow-up time was 2.2 years. The Takotsubo Italian Network (TIN) was the most frequently used database, appearing in two studies with the same time frame (January 2007 to December 2018),^33,34^ suggesting potential overlap and duplication of patient data. Of the two studies, only data from Silverio *et al*.^33^ were included in meta-analysis. Other registries included the InterTAK registry, Mayo Clinic Registry, Kaiser Permanente Southern California Health System (2006–16), and the Spanish National Registry (RETAKO) (January 2003 to July 2018). The included studies employed varying definitions for TTC, largely aligning with established diagnostic criteria from European Society of Cardiology^11^ and INTERTAK.^12^ Almendro-Delia *et al*.^23^ used a modified version of the Mayo Clinic criteria, emphasizing transient wall motion abnormalities beyond a single coronary distribution. Cacciotti *et al*.,^24^ D'Ascenzo *et al*.,^17^ and Elesber *et al*.^26^ described TTC based on transient apical or basal wall motion abnormalities extending beyond a single epicardial coronary territory. Citro 2018^25^ and Lau *et al*.^29^ focused on typical acute presentations involving dyskinesis or akinesis, primarily affecting the apical segments of the left ventricle. Gopalakrishnan *et al*.^27^ defined TTC by transient severe hypokinesis, akinesis, or dyskinesis unrelated to a single coronary distribution, often with stressor-related triggers. The remainder of studies defined TTC according to the INTERTAK registry definition. One study did not use coronary angiogram in the diagnostic algorithm for TTC.^17^ Two studies utilized cardiac MRI in the routine diagnostic algorithm.^24,29^ The weighted mean average mortality rate across the included studies was 4.7%, with 435 patients who died across the included studies. The weighted average recurrence rate across the included studies was ∼3.10%. Of these, 5511 patients (56.59%) were discharged on ACE or ARBs, while 5981 patients (61.47%) received beta-blockers at discharge. Aspirin was prescribed to 4448 patients (45.68%), and statins were given to 2395 patients (24.61%) upon discharge. Characteristics of included studies are detailed in *Table 1*.

**Table 1:** Characteristics of included studies

Study	Number of patients	Follow-up (time points)	Registry used (and year)	Definition of TTC	Diagnosis method	Medication distribution (%)	All-cause mortality (HR 95% CI or event rate)	Primary outcome(s)
Almendro-Delia 2018^23^	711	Median 284 days (IQR: 94–929 days)	RETAKO Registry (2003–16)	Takotsubo syndrome diagnosed based on modified Mayo criteria: transient left ventricular dysfunction (apical, mid-ventricular, basal segmental alterations) extending beyond the territory supplied by a single coronary artery, new ECG changes, absence of myocarditis or pheochromocytoma	Clinical presentation, ECG changes, troponin levels, exclusion of myocarditis/pheochromocytoma, coronary angiography	Beta-blockers 60%, ACE inhibitors/ARBs 62%, statins 50%	Beta-blocker: 0.62 (0.32–0.82) and 6/440	Mortality from cardiovascular (CV) and non-CV causes, TTC recurrence
							ACE/ARB: 0.45 (0.28–0.76) and 7/451	
							Statin: 7/426	
Cacciotti 2012^24^	56	Mean of 2.2 ± 2 years (range 0.1–6.8 years)	Emergency department of Vannini Hospital, Rome, Italy (February 2004 to November 2010)	Transient akinesis or dyskinesis of the LV apical and mid-ventricular segments extending beyond a single vascular distribution	Echocardiography, ECG, coronary angiography, cardiac MR	Aspirin 97.3%, ACE inhibitors/angiotensin receptor blockers 94.6%, β-blockers 89.3%, diuretics 56%, statins 68%	BB: 2/56	LV systolic function recovery
							ACE/ARB: 2/56	
							Aspirin: 2/56	
							Statin: 0/56	
Citro 2018^25^	326	Median 26.5 months	Takotsubo Italian Network (Up to 2019)	Typical transient left ventricular wall motion abnormalities extending beyond a single epicardial vascular distribution with complete functional normalization within 6 weeks, absence of obstructive coronary artery disease	Echocardiography, coronary angiography	β-Blockers 67.9%, ACE inhibitors/angiotensin receptor blockers 70.2%, aspirin 75.9%	ACE/ARB with LVEF < 35%: 16/227	LV systolic function recovery
							No ACE/ARB: 9/99	MACE and rehospitalization
D'Ascenzo 2020^17^	1533	30-day and 5-year	InterTAK Registry (Not specified)	Transient abnormality of left ventricular wall motion beyond a single coronary artery perfusion territory, absence of obstructive coronary artery disease (CAD) or acute plaque rupture	ECG, troponin levels, echocardiography	Aspirin: 67% (1031 out of 1533 patients)	Aspirin: 1.37 (0.79–2.41) and 76/1031	MACCE (major adverse cardiac and cerebrovascular events) at follow-up
							No aspirin: 21/502	
Elesber 2007^26^	100	4 years	Database search at Mayo Clinic (January 1988 through November 2005)	Transient abnormality of left ventricular wall motion with apical hypokinesis, akinesis, or dyskinesis, sparing basal segments	Coronary and left ventricular angiography	Aspirin 74%, beta-blockers 56%	Beta-blocker: 0.80 (0.28–2.29)	All-cause mortality
							ACE or ARB: 1.28 (0.46–3.52)	
							Aspirin: 0.68 (0.24–1.96)	
							Statins: 0.43 (0.10–1.95)	
Gopalakrishnan 2015^27^	56	Mean survival 4.47 years	Study at Advocate Illinois Masonic Medical Center, Chicago (Not specified)	Transient severe hypokinesis, akinesis, or dyskinesis of the left ventricular midsegments with or without apical involvement	Electrocardiogram, echocardiography, coronary angiography	Aspirin 73.2%, beta-blockers 82.1%, ACE inhibitors 83.9%, statins 67.9%	Beta-blocker: 0.51 (0.04–6.36) and 10/46	Repeat LVEF evaluation showing improvement, survival analysis
							ACE or ARB: 1.33 (0.11–16.37) and 10/47	
							Aspirin: 0.94 (0.20–4.40) and 8/41	
							Statins: 0.62 (0.15–2.58) and 7/38	
Kim 2018^28^	265	Median follow-up of 5.8 years	Mayo Clinic Takotsubo Syndrome Registry (January 2002 through December 2016)	Transient wall motion abnormalities not confined to a single vascular territory with normal coronaries	ECG, echocardiography, coronary angiography	Beta-blockers 89%, ACE inhibitors 73%, aspirin 66.7%	Aspirin: 0.94 (0.428–1.342)	MACCE including all-cause mortality, non-fatal MI, stroke, hospitalization for heart failure
							ACE: 1.06 (0.680–1.573)	
							Statin: 1.034 (0.680–1.573)	
							Beta-blocker: 0.758 (0.428–1.342)	
							8 deaths during hospitalization	
Lau 2021^29^	519	Median of 5.2 years (IQR 3.0–7.2 years)	Kaiser Permanente Southern California Health System (2006–16)	Typical acute transient left ventricular dysfunction related to stress, not related to coronary artery occlusion	Coronary angiography, cardiac imaging	Beta-blockers 86.1%, ACE inhibitors/ARBs 77.5%	Beta-blocker: 0.46 (0.29–0.72) and 58/447	All-cause mortality
							ACE/ARB: 0.92 (0.59–1.42) and 56/402	
							Mineralocorticoid receptor antagonist: 1.42 (0.66–3.05) and 7/23	
								TTC Recurrence
Novo 2023^30^	2429	Median of 1 year (386 days)	GEIST (German Italian Spanish Takotsubo) registry (2002–17)	Takotsubo syndrome diagnosed according to Heart Failure Association Criteria	Echocardiography, coronary angiography	Statins 53.2%, non-statins 46.8%	ACE or ARB: 0.604 (0.433–0.844)	All-cause mortality
							Beta-blocker: 0.880 (0.653–1.185)	
							Statin: 0.966 (0.738–1.265) and 115/1267	
Pereyra 2021^31^	544	Median 25 months, up to 2 years	Spanish National Registry (RETAKO) (June 2002 to March 2017)	Characterized by transient apical ballooning of the left ventricle, presenting with symptoms and electrocardiographic signs mimicking acute coronary syndrome, absence of pheochromocytoma, myocarditis, or hypertrophic cardiomyopathy	Electrocardiography, echocardiography, coronary angiography	Aspirin 72%, P2Y12 inhibitors used less frequently	Antiplatelet: HR 0.315; 95% CI 0.106–0.943	Major adverse cardiovascular adverse events
Raposeiras-Roubin 2023^32^	970	Mean 2.5 ± 3.3 years	Spanish National Registry (RETAKO) (January 2003 to July 2018)	Diagnosed according to modified Mayo criteria, transient left ventricular dysfunction with apical, mid-ventricular, or basal segmental alterations extending beyond the territory supplied by a single coronary artery	Echocardiography, coronary angiography, ECG	Beta-blockers 60%	Beta-blocker: 0.95, 95% CI 0.57–1.13. Mortality rate 3.3 (95% CI 2.4–4.3) per 100 patients/year	All-cause mortality
								TTC recurrence
Silverio 2022^33^	825	Median 24 months	Takotsubo Italian Network (TIN) (January 2007 to December 2018)	Takotsubo syndrome diagnosed according to the TIN, Heart Failure Association and InterTAK Diagnostic Criteria, transient left ventricular dysfunction	Echocardiography, coronary angiography, ECG	Beta-blockers 59.2%	Beta-blocker: HR: 0.563 (0.356–0.889) and 33/448	Long-term survival
Silverio 2023^34^	903	Median 24 months (IQR 11–38 months)	Takotsubo Italian Network (TIN) (January 2007 to December 2018)	Takotsubo syndrome diagnosed according to TIN and Heart Failure Association criteria, featuring transient left ventricular dysfunction with apical ballooning, absence of coronary artery disease	Echocardiography, coronary angiography, ECG	Beta-blockers 60.7%, RAAS inhibitors 41.1%	*Beta-blocker: 0.59 (0.39–0.89)	Composite of all-cause death and TTC recurrence
							ACE or ARB 0.79 (0.51–1.21)	
							*Due to overlapping data with Silverio 2022, only the ACE or ARB hazard ratio was included in meta-analysis	

### Baseline characteristics

The patient sample size added up to a total of 9237 patients. The average age was 69.7 ± 12.5 years. Among included studies, 7906 (90.36%) were female. Anatomical variants were reported in only one study.^24^ A total of 1026 patients (34.39%) experienced a physical stressor preceding their admission with TTC. Of the included patients, 23.1% had known coronary artery disease, 17.9% had diabetes mellitus, 44.8% had dyslipidaemia, and 65.8% had hypertension. The average LVEF across all studies was 41.12% (±10.54%), and the average QTc was 489.33 ms (±51.75 ms). The total number of patients with LVOTO was 11 (0.11%). Detailed baseline characteristics are provided in the supplementary materials.

### Methodological quality and risk of bias

The methodological quality of the included studies was generally moderate, as assessed by the ROBINS-I tool. Across the 13 studies, the overall risk of bias was consistently rated as moderate, primarily due to the observational nature of the designs, which inherently carried risks of confounding and missing data. The domain most frequently associated with higher bias was confounding, present in all studies. This was attributed to the diverse patient populations, varying management strategies, and differences in healthcare settings. Despite adjustment efforts, unmeasured or residual confounding persisted, particularly in multicentre registry-based studies like Almendro-Delia *et al*.^23^ and Raposeiras-Roubín *et al*.^32^ where local practice variations contributed to bias. Additionally, bias due to missing data was common across all studies, with many not clearly specifying strategies for managing missing outcomes, especially in those with longer follow-up periods. Conversely, bias in selection of participants, classification of interventions, measurement of outcomes, and selection of reported results were generally low. Most studies used clear inclusion criteria, consistent intervention definitions, and objective outcomes like mortality and recurrence rates, reducing bias. Moreover, adherence to predefined protocols minimized selective reporting. Among the studies, Almendro-Delia *et al*.^23^ and D'Ascenzo *et al*.^17^ exhibited relatively higher bias, particularly due to confounding and inconsistent handling of missing data. These studies, often based on larger multicentre registries or cohort analyses, faced issues with data completeness and selection bias. In contrast, studies like Kim *et al*.^28^ and Silverio *et al*.^33^ showed lower bias within the moderate range, due to advanced statistical analysis and adjustment of confounders. A detailed risk of bias summary is provided in the supplementary materials.

### Association of outcomes with secondary prevention medication according to the Bayesian approach

**Figure 2:**
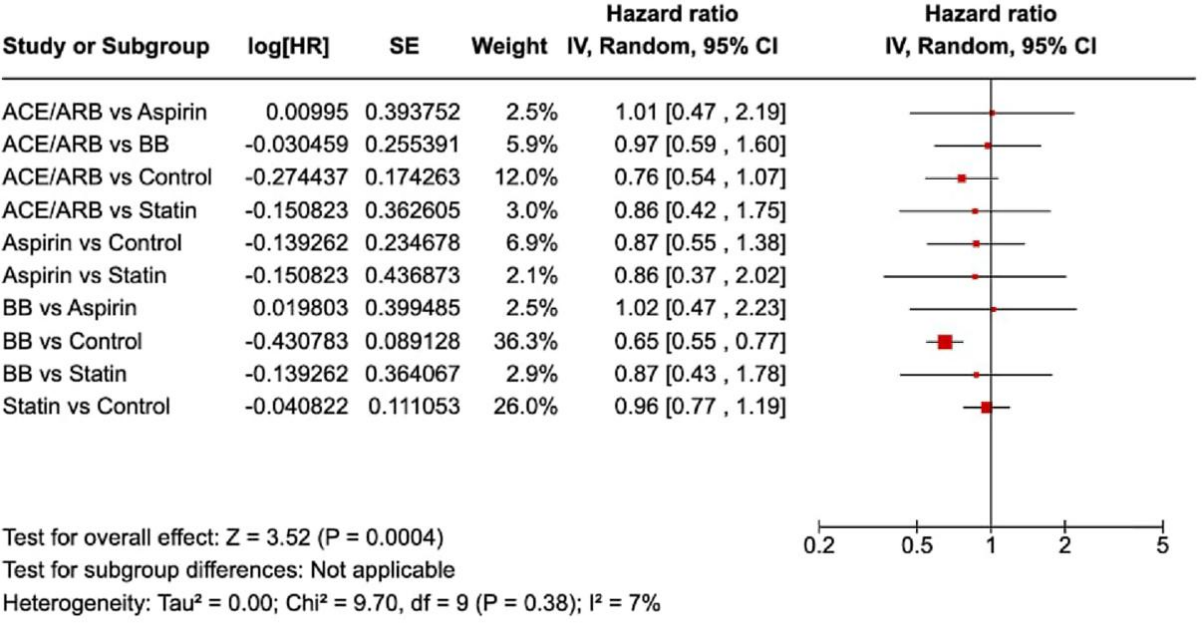
Forest plot comparing treatment arms [ACE/ARB, beta-blocker (BB), aspirin, statin] with hazard ratio (HR, 95% confidence intervals) for all-cause mortality.

**Figure 3:**
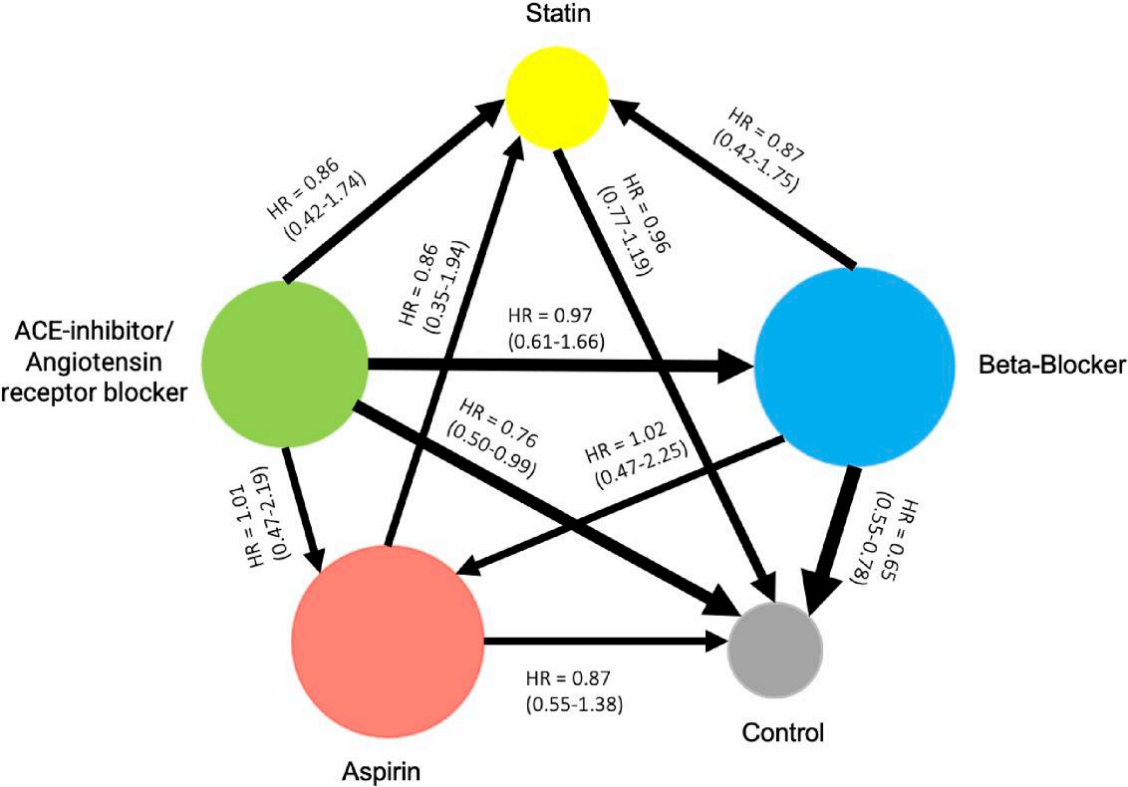
Network diagram comparing survival benefit of medical therapy in takotsubo cardiomyopathy. Angiotensin-converting enzyme inhibitors or angiotensin receptor blockers (ACE/ARB), aspirin, statins, beta-blockers, and control (no pharmacotherapy) are represented as nodes. The size of each node reflects the number of patients who received that treatment across the included studies. Lines between nodes represent direct comparisons between two treatments, with the width proportional to the number of contributing studies. Arrows indicate the direction of comparison, pointing from the reference treatment to the comparator. Hazard ratios (HRs) and 95% confidence intervals (CIs) are displayed on the arrows and reflect the relative hazard of mortality for the comparator compared to the reference treatment. For example, the arrow from ACE/ARB to beta-blockers displays an HR of 0.86 [95% CI (0.48–1.51)], indicating that, in the studies that directly compared these two treatments, patients who received beta-blockers had a numerically lower—but not statistically significant—hazard of mortality compared to those who received ACE/ARB therapy. An HR < 1.0 favours the comparator (in this case, beta-blockers), while an HR > 1.0 would favour the reference treatment. The absence of a connecting line between two nodes indicates that no direct comparison was available in the included studies.

ACE/ARB treatment showed a reduction in mortality risk compared to control [HR = 0.76, 95% CI (0.54–1.07)]. However, this was not statistically significant. BB therapy was associated with a significantly lower risk of all-cause mortality compared to control [HR = 0.65, 95% CI (0.55–0.77)]. Statin therapy did not show a significant difference in mortality risk compared to control [HR = 0.96, 95% CI (0.77–1.19)]. Aspirin use was associated with a nonsignificant trend towards reduced mortality [HR = 0.87, 95% CI (0.55–1.38)]. BB vs. statins [HR = 0.87, 95% CI (0.43–1.78)] and BB vs. aspirin [HR = 1.02, 95% CI (0.47–2.23)] indicated no significant differences. *Figure 2* displays a summary forest plot comparing treatment arms [ACE/ARB, beta-blocker (BB), aspirin, statin] with hazard ratio (HR, 95% CIs) for all-cause mortality. A graphical representation of the association of secondary prevention medication with TTC mortality and recurrence is displayed in *Figure 3*. The league table is shown in *Table 2*.

**Table 2:** League table comparing survival benefit of medical therapy in takotsubo cardiomyopathy

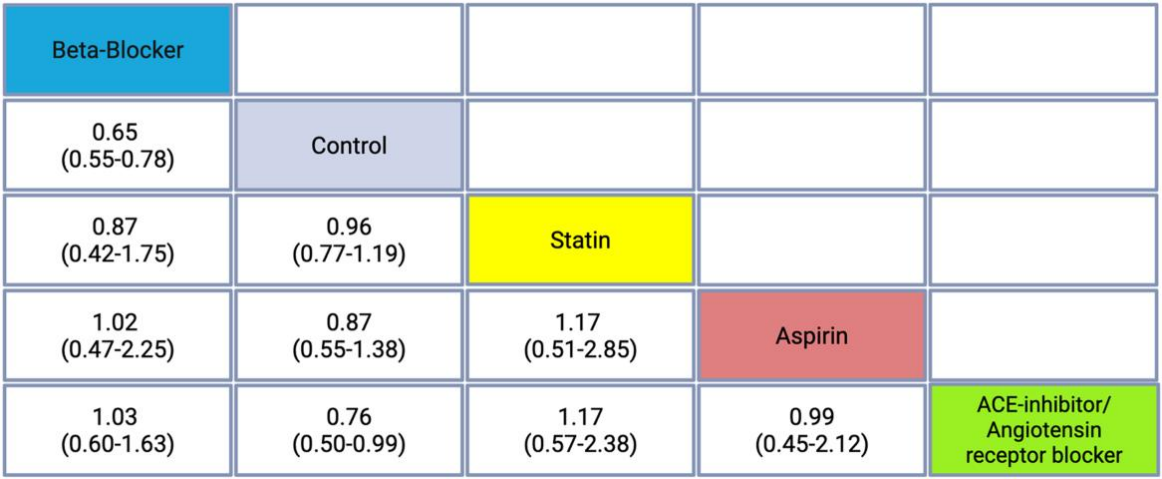

The table presents hazard ratios (HRs) and 95% confidence intervals (CIs) for each pairwise comparison of medical therapies. Each row defines the reference treatment, and each column the comparator. A hazard ratio (HR) < 1 suggests lower hazard of mortality with the row treatment compared to the column treatment; an HR > 1 favours the column treatment. For example, in the row for beta-blockers, the comparison with control indicates a hazard ratio of 0.65 [95% CI (0.55–0.77)], suggesting a lower mortality risk associated with beta-blockers.

The probabilities of each treatment being ranked from most to least effective (Rank 1 to Rank 4) were analysed alongside the Surface Under the Cumulative Ranking (SUCRA) values. ACE/ARBs showed a 14% probability of being the most effective treatment, a 27% probability for both the second and fourth most effective, and a 32% probability for the third most effective, with a SUCRA value of 42.97. Aspirin had a 23% probability of being the most effective, 22% for second, 19% for third, and 36% for fourth, resulting in a SUCRA of 44.02. Beta-Blockers displayed a 17% chance of being ranked first, 28% second, 33% third, and 22% fourth, with a SUCRA of 47.14. Statins were found to be the most favourable treatment with a 45% probability of being the most effective, 23% for second, 17% for third, and 15% for fourth, accompanied by the highest SUCRA value of 65.87. Rankograms and SUCRA values are detailed in *Table 3*.

**Table 3:** Rankogram and SUCRA values

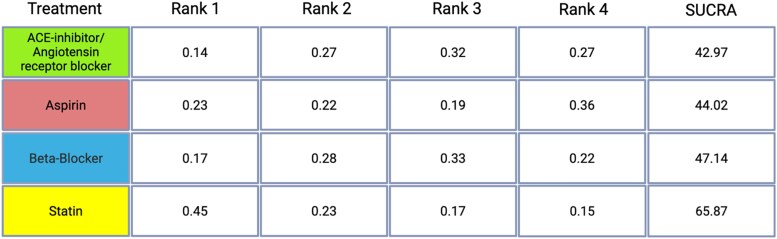

The table presents the probability of each treatment being ranked from first (most effective) to fourth (least effective) for mortality reduction, along with the Surface Under the Cumulative Ranking curve (SUCRA) values. Each row corresponds to a treatment, and columns indicate the probability of occupying each rank. SUCRA values summarize these probabilities into a single metric ranging from 0% to 100%, where higher values indicate a greater likelihood of being among the more effective treatments. A SUCRA of 90% implies that a treatment achieves 90% of the maximum possible effectiveness relative to others.

## Discussion

The present study of 9237 patients found that:

- Rate of TTC mortality and recurrence is 4.7% and 3.10%, respectively, at average 2.2 years follow-up.
- Single-drug therapy with beta-blockers (BB) was associated with 35% reduction in TTC mortality, but failed to demonstrate significant incremental survival benefit on comparison with ACE/ARB, aspirin, and statin.
- Single-drug therapy with ACE/ARB, aspirin, or statin was not associated with reduction of TTC mortality, and no medications demonstrated significant incremental improvement in direct comparison.
- There is not sufficient statistical evidence regarding therapy with secondary prevention medical therapy in reduction of TTC mortality.

This meta-analysis evaluated the association between secondary prevention therapies (ACE/ARB, beta-blockers, aspirin, and statins) and all-cause mortality in TTC patients. Recent studies have shown that TTC has morbidity and mortality rates that are comparable to those of ACS, with ∼40% of all long-term death in these patients related specifically to TTC-related complications.^8–13^ The rate of TTC mortality reported in our pooled study (4.7%) was consistent with the International Takotsubo Registry^12^ and previous meta-analyses.^8^ Previous studies have investigated the association between medical therapy and TTC recurrence, and found no significant reduction in TTC recurrence with ACE/ARB, BB, the combination of both medications, and no incremental reduction in TTC recurrence after direct comparison.^16,35^ Prior meta-analyses evaluating survival benefit have faced various limitations: excluding patients from the International Takotsubo Registry, inclusion of unadjusted observational studies, and pooling of distinct risk estimates.^12,36,37^ Pooled analyses have reported a medication distribution at index admission for BB of 66.8% and ACE/ARB of 67.4%, and the International Takotsubo Registry reports on admission, 32.5% of the patients were taking BB and 37.9% were taking either ACE/ARB, which increased at discharge to 78.1% and 79.3%, respectively. This is complemented by the results from the current study, which reported 61.47% for BB and 56.59% for ACE/ARB on discharge. The distribution of patients discharged on aspirin or statins is more inconsistent, however the presence of concomitant CAD in TTC patients has been reported to be 10–12%, which is less than that of the current review (23.1%). Thus, the novelty of our study is that it (i) evaluates the association of medical therapy with TTC mortality, which is especially pertinent given the comparable mortality rates to ACS,^8^ as well as similar observed incidence to TTC recurrence,^16,38^ (ii) includes a larger sample size than previous meta-analyses, (iii) employs hierarchical Bayesian network meta-analysis, evaluating the relative, incremental mortality benefit of medical therapies while incorporating prior probabilities and generating posterior distributions, and (iv) employed SUCRA rankograms to provide a probabilistic estimation of treatment effectiveness, improving interpretability of the findings.^22^

### Angiotensin-converting enzyme inhibitors and angiotensin receptor blockers

ACE/ARBs have been hypothesized to reduce mortality in TTC by attenuating sympathetic overactivity and reducing afterload, which are critical contributors to ventricular stress and myocardial stunning observed in TTC​.^5^ The potential benefit of ACE inhibitors is thought to arise from their capacity to mitigate the effects of excessive catecholamine release on the myocardium, which includes reducing myocardial oxygen demand and improving endothelial function, as well as slowing the progression to LV dysfunction by improving ventricular remodelling and reducing myocardial fibrosis.^5,39^ Mechanistic studies supporting the use of ACE inhibitors in TTC postulate an improvement in coronary microvascular function, which is often impaired in TTC patients due to endothelial dysfunction and microvascular spasm triggered by catecholamine excess.^39^ In our study, although ACE/ARB therapy was associated with a reduction in mortality risk [HR = 0.76, 95% CI (0.54–1.07)], this finding did not achieve statistical significance, notably limited by heterogeneity in baseline demographics and underpowered analysis. Of note, ACE and ARBs have demonstrated mortality benefit in a large propensity-matched study based on the InterTAK registry, suggesting they may play a supportive role in facilitating LV recovery, especially in patients with severe LV dysfunction or high systemic vascular resistance.^11,12^

### Beta-blockers

Given the elevated catecholamine levels from trigger events observed in TTC, beta-blockers have been postulated to have a role in the long-term management of TTC until full recovery of LVEF is achieved, although clinical trials supporting this approach are lacking.^33^ Plasma catecholamine levels in patients with TTC increase to three times higher than the catecholamine levels in patients with acute myocardial infarction and postinfarction heart failure.^40^ As such, BB therapy may attenuate the myocardial damage caused by sympathetic overstimulation resulting from catecholamine surge from trigger events. Animal studies have demonstrated that apical ballooning can be mitigated with drugs possessing both alpha and beta-adrenoceptor blocking properties, where intravenous metoprolol has been shown to improve epinephrine-induced apical ballooning.^41,42^ Beta-blockers have demonstrated inconsistent mortality benefit in the literature.^12,37^ The lack of specific guideline recommendation for long-term beta-blockers after TTC recovery reflects the variable prescribing patterns in global registries.^11,43^ The pooled proportion of patients discharged on beta-blockers in our review (61.47%) was similar to that reported in the large multicentre RETAKO registry^23^ (60%) but lower than the InterTAK registry (78%). Our study demonstrated long-term survival benefit for beta-blockers compared to control. These results should be interpreted with caution and are largely exploratory due to the inclusion of underpowered studies and the notable exclusion of a large propensity-matched study by Templin *et al*. from the InterTAK registry^11^ as there were insufficient data to calculate HRs. This study notably failed to demonstrate long-term survival benefit for beta-blockers, and its exclusion represents inherent limitations of meta-analysis; including the use of cumulative data from summary estimates rather than individual patient data and follow-up. The results of our study are thus similar to a recent meta-analysis evaluating the long-term mortality benefit of beta-blockers^37^ [pooled HR = 0.62; 95% CI (0.50–0.79)], which also excluded the InterTAK study. We hypothesize that inclusion of this study may alter the findings for the following reasons; (i) the study by Templin *et al*.^5^ included a broader and more heterogeneous patient population with a greater proportion of patients with severe emotional and physical stressors (27.7% and 36.0%, respectively) and comorbidities, which may have diluted the treatment effect of beta-blockers in the pooled analysis, (ii) differences in average follow-up, and (iii) unmeasured confounding due to overlapping medication use as not all studies in the meta-analysis disclosed the isolated treatment effects of beta-blockers and inconsistently reported distribution of ‘severe TTC’ and incidence of cardiogenic shock, LVOTO > 40 mmHg, and VT. Due to inconsistent reporting of stressors across all included studies, we were unable to perform subgroup analysis for ‘secondary’ TTC, which may have further defined the role of BB in the management of TTC. Importantly, the rationale behind discharging patients on beta-blockers was not clear for all studies. This can be influenced by perceived angina control in patients with concomitant CAD, and discouraged in patients with chronic obstructive pulmonary disease, as well as haemodynamic status (blood pressure, heart rate, acute heart failure, cardiogenic shock requiring intra-aortic balloon pump, and inotropes).

### Aspirin and statins

Aspirin's role in TTC management has been linked to its potential antithrombotic effect, given the increased platelet activation and endothelial dysfunction associated with TTC pathogenesis.^44^ Prolonged catecholamine exposure in TTC is thought to trigger platelet aggregation, suggesting that aspirin could mitigate thromboembolic complications.^45^ In the current meta-analysis, aspirin was associated with a nonsignificant trend towards mortality reduction, highlighting its limited efficacy in this population. While observational studies have shown that antiplatelet therapy at discharge may reduce early adverse outcomes,^46^ evidence for its long-term benefit remains inconclusive, and aspirin use should be considered on a case-by-case basis, particularly for patients with concomitant CAD. Statins have been postulated to reduce TTC recurrence by reducing microvascular dysfunction and the oxidative stress associated with TTC.^5,30^ As hypothesized, these theoretical effects did not translate to a significant mortality benefit. In contrast to ACS, TTC is primarily characterized by catecholamine-induced myocardial stunning, microvascular dysfunction, and transient left ventricular wall motion abnormalities, often without significant epicardial coronary obstruction.^5^

### Limitations

This meta-analysis faces several limitations inherent to the available evidence. Our findings are largely exploratory since many studies included in the review lack sufficient statistical power to detect differences in hard clinical endpoints. All included studies were observational, which significantly raises concerns for unmeasured confounding, selection bias, and publication bias. Additionally, the assumption of transitivity in network meta-analysis may be challenged by heterogeneity across studies in terms of population characteristics, treatment definitions, follow-up durations, and statistical adjustments. Inconsistent reporting of medication dosing, overlapping medication regimens, combination therapies, precise timing of commencement and duration of therapy as well as adverse effects/intolerances, likely diluted the inter-group comparisons. Importantly, the rationale behind discharging the patients on each medication group was inconsistently disclosed. Substantial heterogeneity was observed in patient characteristics, ranging from primary to secondary TTC phenotypes, physical and emotional stressors, and variations in LVOTO and LVEF. The evolving diagnostic criteria for TTC further complicate the interpretation of pooled results, as some included cases may represent alternative diagnoses. Despite a generally high methodological quality among the studies, inherent heterogeneity regarding TTC aetiology remains a challenge, compounded by inconsistent use of coronary angiograms and cardiac MRI, potentially leading to misclassification of non-ischaemic myocardial injuries as TTC. The median follow-up of 2.2 years may underestimate the frequency of late events associated with secondary prevention medical therapy. Notably, as a limitation of meta-analysis, we were unable to include all studies for every outcome parameter investigated. Consequently, the number of the patients entering the meta-analysis varies greatly, generally constituting only a fraction of the whole study population.

### Implications for future research

The current meta-analysis establishes significant knowledge gaps in the medical management of TTC. Despite the prevalence, morbidity, and mortality associated with TTC, the lack of statistical power and heterogeneity of existing data necessitates higher-quality evidence through RCT evaluation of the differential treatment effects of ACE/ARB, BB, aspirin, and statins. It is hoped that such evidence will be provided by the upcoming NACRAM (*n*-Acetylcysteine and Ramipril Takotsubo Syndrome Trial, ACTRN12616000781448) and BROKEN-SWEDEHEART [Optimized Pharmacological Treatment for Broken Heart (Takotsubo) Syndrome, NCT04666454], and Beta-Blockers in Takotsubo Syndrome Study (β-Tako, NCT06509074) trials. Machine-learning approaches may enhance TTC prognostication by identifying phenotypic clusters with distinct risk profiles. Integrating these methods into future studies could further tailor patient management strategies and clarify the role of secondary prevention therapies in high-risk subgroups.^47^ Research into the effects of stress and adrenergic stimulation on the heart and other organs is extensive, but the impact of elevated catecholamines remains less understood. High stress and catecholamine levels affect cardiovascular function through various mechanisms, including receptor dynamics, signalling pathways, mitochondrial activity, inflammation, metabolism, and gene expression. Previous studies have reported reduction in ‘all-cause’ and cardiac mortality in patients treated with ACE or ARB, and beta-blockers,^33^ however we were unable to pool separate results in the current meta-analysis due to inconsistent data with many studies not powered to detect mortality. As such, the cardiac/non-cardiac mortality ratio, the exact influence of pharmacotherapy on non-cardiac mortality, and why TTC patients remain at risk of long-term mortality remain uncertain.

## Conclusions

This review highlights the modest efficacy of secondary prevention medications in the management of TTC. Randomized controlled trials are necessary to define the treatment effects of pharmacotherapy in this vulnerable patient cohort.

## Supplementary Material

oeaf040_Supplementary_Data

## Data Availability

The data underlying this article are available in the article and in its online Supplementary material.

## Lead author biography

Dr Daud Mutahar is a Junior Doctor at Gold Coast University Hospital, Australia. He is an early-career researcher with a strong interest in evidence-based medicine and improving patient outcomes through clinical practice and academic work.

Dr Ammar Zaka is a Basic Physician Trainee at Gold Coast University Hospital, Australia, with clinical and research interests in cardiovascular medicine.
