# Supplementary material for: Survival benefit of secondary prevention medical therapy in takotsubo cardiomyopathy: a Bayesian network meta-analysis
Source: Eur Heart J Open. 2025 Apr 16;5(3):oeaf040. doi: 10.1093/ehjopen/oeaf040 (PMC12066946; doi:10.1093/ehjopen/oeaf040)
Supplement: oeaf040_Supplementary_Data [file oeaf040_supplementary_data.docx]

**SUPPLEMENTARY DIGITAL CONTENT**

**List of Supplementary Digital Content**

- Appendix 1: PRISMA 2020 Checklist
- Appendix 2: MOOSE
- Appendix 3: Search String
- Appendix 4: Justification of exclusions at full-text review
- Appendix 5: Risk of bias assessment for included studies using the ROBINS-I risk of bias tool
- Appendix 6: Baseline Demographics (excel sheet) and Figure 4: Extended Forest Plot of all studies including in meta-analysis

**Appendix 1:**

**Supplementary Table 1 - PRISMA 2020 Checklist**

| **Section and Topic** | **Item #** | **Checklist item** | **Location where item is reported** |
| --- | --- | --- | --- |
| **TITLE** | | |  |
| Title | 1 | Identify the report as a systematic review. | Title page |
| **ABSTRACT** | | |  |
| Abstract | 2 | See the PRISMA 2020 for Abstracts checklist. | 3 |
| **INTRODUCTION** | | |  |
| Rationale | 3 | Describe the rationale for the review in the context of existing knowledge. | 4-5 |
| Objectives | 4 | Provide an explicit statement of the objective(s) or question(s) the review addresses. | 4 |
| **METHODS** | | |  |
| Eligibility criteria | 5 | Specify the inclusion and exclusion criteria for the review and how studies were grouped for the syntheses. | 5 |
| Information sources | 6 | Specify all databases, registers, websites, organisations, reference lists and other sources searched or consulted to identify studies. Specify the date when each source was last searched or consulted. | 5 |
| Search strategy | 7 | Present the full search strategies for all databases, registers and websites, including any filters and limits used. | 5, Supp. p.7 |
| Selection process | 8 | Specify the methods used to decide whether a study met the inclusion criteria of the review, including how many reviewers screened each record and each report retrieved, whether they worked independently, and if applicable, details of automation tools used in the process. | 5-6 |
| Data collection process | 9 | Specify the methods used to collect data from reports, including how many reviewers collected data from each report, whether they worked independently, any processes for obtaining or confirming data from study investigators, and if applicable, details of automation tools used in the process. | 5-6 |
| Data items | 10a | List and define all outcomes for which data were sought. Specify whether all results that were compatible with each outcome domain in each study were sought (e.g. for all measures, time points, analyses), and if not, the methods used to decide which results to collect. | 5-6 |
|  | 10b | List and define all other variables for which data were sought (e.g. participant and intervention characteristics, funding sources). Describe any assumptions made about any missing or unclear information. | 5-6 |
| Study risk of bias assessment | 11 | Specify the methods used to assess risk of bias in the included studies, including details of the tool(s) used, how many reviewers assessed each study and whether they worked independently, and if applicable, details of automation tools used in the process. | 6 |
| Effect measures | 12 | Specify for each outcome the effect measure(s) (e.g. risk ratio, mean difference) used in the synthesis or presentation of results. | 5 |
| Synthesis methods | 13a | Describe the processes used to decide which studies were eligible for each synthesis (e.g. tabulating the study intervention characteristics and comparing against the planned groups for each synthesis (item #5)). | 5-6 |
|  | 13b | Describe any methods required to prepare the data for presentation or synthesis, such as handling of missing summary statistics, or data conversions. | 5-6 |
|  | 13c | Describe any methods used to tabulate or visually display results of individual studies and syntheses. | 5-6 |
|  | 13d | Describe any methods used to synthesize results and provide a rationale for the choice(s). If meta-analysis was performed, describe the model(s), method(s) to identify the presence and extent of statistical heterogeneity, and software package(s) used. | 6 |
|  | 13e | Describe any methods used to explore possible causes of heterogeneity among study results (e.g. subgroup analysis, meta-regression). | 6 |
|  | 13f | Describe any sensitivity analyses conducted to assess robustness of the synthesized results. | 6 |
| Reporting bias assessment | 14 | Describe any methods used to assess risk of bias due to missing results in a synthesis (arising from reporting biases). | 6 |
| Certainty assessment | 15 | Describe any methods used to assess certainty (or confidence) in the body of evidence for an outcome. | 5-6 |
| **RESULTS** | | |  |
| Study selection | 16a | Describe the results of the search and selection process, from the number of records identified in the search to the number of studies included in the review, ideally using a flow diagram. | 7- 9 (Figure 2,3). Tables 2-3 |
|  | 16b | Cite studies that might appear to meet the inclusion criteria, but which were excluded, and explain why they were excluded. | 19 (Figure 1), Supp. p.8-25 |
| Study characteristics | 17 | Cite each included study and present its characteristics. | 22-23 (Table 1) |
| Risk of bias in studies | 18 | Present assessments of risk of bias for each included study. | 8, Supp. p26 |
| Results of individual studies | 19 | For all outcomes, present, for each study: (a) summary statistics for each group (where appropriate) and (b) an effect estimate and its precision (e.g. confidence/credible interval), ideally using structured tables or plots. | 7-8, 20-21 (Figure 2 and 3) |
| Results of syntheses | 20a | For each synthesis, briefly summarise the characteristics and risk of bias among contributing studies. | 22-23 (Table 1), Supp. p26 |
|  | 20b | Present results of all statistical syntheses conducted. If meta-analysis was done, present for each the summary estimate and its precision (e.g. confidence/credible interval) and measures of statistical heterogeneity. If comparing groups, describe the direction of the effect. | 7-8, 20-21 (Figure 2 and 3) |
|  | 20c | Present results of all investigations of possible causes of heterogeneity among study results. | 6 |
|  | 20d | Present results of all sensitivity analyses conducted to assess the robustness of the synthesized results. | 7-8 |
| Reporting biases | 21 | Present assessments of risk of bias due to missing results (arising from reporting biases) for each synthesis assessed. | 7, Supp material |
| Certainty of evidence | 22 | Present assessments of certainty (or confidence) in the body of evidence for each outcome assessed. | 7-9, |
| **DISCUSSION** | | |  |
| Discussion | 23a | Provide a general interpretation of the results in the context of other evidence. | 10-13 |
|  | 23b | Discuss any limitations of the evidence included in the review. | 10-13 |
|  | 23c | Discuss any limitations of the review processes used. | 10-13 |
|  | 23d | Discuss implications of the results for practice, policy, and future research. | 10-13 |
| **OTHER INFORMATION** | | |  |
| Registration and protocol | 24a | Provide registration information for the review, including register name and registration number, or state that the review was not registered. | 5 |
|  | 24b | Indicate where the review protocol can be accessed, or state that a protocol was not prepared. | 5 |
|  | 24c | Describe and explain any amendments to information provided at registration or in the protocol. | Not applicable |
| Support | 25 | Describe sources of financial or non-financial support for the review, and the role of the funders or sponsors in the review. | Title page |
| Competing interests | 26 | Declare any competing interests of review authors. | Title page |
| Availability of data, code and other materials | 27 | Report which of the following are publicly available and where they can be found: template data collection forms; data extracted from included studies; data used for all analyses; analytic code; any other materials used in the review. | Title page |

**Appendix 2**

**Supplementary Table 2: MOOSE Checklist**

| **Reporting Criteria** | **Reported (Yes/No)** | **Reported on Page Number** |
| --- | --- | --- |
| Problem definition | Yes | 2-3 |
| Hypothesis statement | Yes | 3 |
| Description of study outcomes | Yes | 5 |
| Type of exposure or intervention used | Yes | 5 |
| Type of study designs used | Yes | 5 |
| Study population | Yes | 5 |
| Qualifications of searchers (e.g. librarians and investigators) | Yes | Title |
| Search strategy, including time period included in the synthesis and keywords | Yes | 5, Supp. p7 |
| Effort to include all available studies, including contact with authors | Yes | 5-6 |
| Databases and registries searched | Yes | 5 |
| Search software used, name and version, including special features used (e.g. explosion) | Yes | 5 |
| Use of hand searching (e.g. reference lists of obtained articles) | Yes | 5 |
| List of citations located and those excluded, including justification | Yes | 5, 19, Supp. p8-25 |
| Methods of addressing articles published in languages other than English | Yes | 5 |
| Method of handling abstracts and unpublished studies | Yes | 5 |
| Description of any contact with authors | Yes | 5 |
| Description of relevance or appropriateness of studies assembled for assessing the hypothesis to be tested | Yes | 5-6 |
| Rationale for the selection and coding of data (e.g. sound clinical principles or convenience) | Yes | 5-6 |
| Documentation of how data were classified and coded (e.g. multiple raters, blinding, and interrater reliability) | Yes | 5-6 |
| Assessment of confounding (e.g. comparability of cases and controls in studies where appropriate) | No |  |
| Assessment of study quality, including blinding of quality assessors; stratification or regression on possible predictors of study results | Yes | 5-6 |
| Assessment of heterogeneity | No |  |
| Description of statistical methods (e.g. complete description of fixed or random effects models, justification of whether the chosen models account for predictors of study results, dose-response models, or cumulative meta-analysis) in sufficient detail to be replicated | Yes | 5-7 |
| Provision of appropriate tables and graphics | Yes | Table 1, Figure 1, 2 and 3 |
| Graphic summarizing individual study estimates and overall estimate | Yes | Figure 1 |
| Table giving descriptive information for each study included | Yes | Table 1 |
| Results of sensitivity testing (e.g. subgroup analysis) | Yes | 8, Figure 3 |
| Indication of statistical uncertainty of findings | Yes | 7-8, Figure 2 and 3 |
| Quantitative assessment of bias (e.g. publication bias) | Yes | 7 |
| Justification for exclusion (e.g. exclusion of non-English-language citations) | Yes | Supp. |
| Assessment of quality of included studies | Yes | 7, Supp |
| Consideration of alternative explanations for observed results | Yes | 9-11 |
| Generalization of the conclusions (i.e. appropriate for the data presented and within the domain of the literature review) | Yes | 9-11 |
| Guidelines for future research | Yes | 9-11 |
| Disclosure of funding source | Yes | Title Page |

**Appendix 3:**

**Supplementary Table 3 – Search String**

| **Number** | **Search Terms** | **Pubmed** | **EMBASE** | **Cochrane** |
| --- | --- | --- | --- | --- |
| **1** | ((Takotsubo Cardiomyopathy[MeSH Terms]) OR (Takotsubo Syndrome[Title/Abstract]) OR (TTS[Title/Abstract]) OR (apical ballooning syndrome[Title/Abstract]) OR (broken heart syndrome[Title/Abstract]) OR (stress cardiomyopathy[Title/Abstract])) | **9329** | **18197** | **644** |
| **2** | ((Prognosis[Title/Abstract]) OR (Recurrence[Title/Abstract]) OR (Mortality[Title/Abstract]) OR (Survival[Title/Abstract}) OR (Pharm*[Title/Abstract])) | **3,044,806** | **5159459** | **290597** |
| **3** | 1 AND 2 | **1,590** | **4705** | **72** |

**Total (after removal of duplicates) = 2502**

**Full text review = 34**

**Appendix 4: Justification of Exclusions at Full-Text Review**

**Excluded Studies (n=21)**

**Wrong study design (n=5)**

1. Akashi YJ, Nef HM, Lyon AR. Epidemiology and pathophysiology of Takotsubo syndrome. Nat Rev Cardiol. 2015;12(7):387-97. Epub 20150407. doi: 10.1038/nrcardio.2015.39. PubMed PMID: 25855605.
2. El-Battrawy I, Santoro F, Stiermaier T, Möller C, Guastafierro F, Novo G, Novo S, Mariano E, Romeo F, Romeo F, Thiele H, Guerra F, Capucci A, Giannini I, Brunetti ND, Eitel I, Akin I. Incidence and Clinical Impact of Recurrent Takotsubo Syndrome: Results From the GEIST Registry. J Am Heart Assoc. 2019;8(9):e010753. doi: 10.1161/jaha.118.010753. PubMed PMID: 31046506; PMCID: PMC6512083.
3. Gupta S, Gupta MM. Takotsubo syndrome. Indian Heart J. 2018;70(1):165-74. Epub 20170913. doi: 10.1016/j.ihj.2017.09.005. PubMed PMID: 29455773; PMCID: PMC5902911.
4. Mugnai G, Pasqualin G, Prati D, Menegatti G, Vassanelli C. Recurrent multiform Takotsubo cardiomyopathy in a patient with epilepsy: Broken heart or brain? Int J Cardiol. 2015;201:332-5. Epub 20141127. doi: 10.1016/j.ijcard.2014.11.212. PubMed PMID: 26301636.
5. Pelliccia F, Pasceri V, Patti G, Tanzilli G, Speciale G, Gaudio C, Camici PG. Long-Term Prognosis and Outcome Predictors in Takotsubo Syndrome: A Systematic Review and Meta-Regression Study. JACC Heart Fail. 2019;7(2)

**No comparator (n=7)**

1. Bento D, Azevedo O, Santos R, Almeida A, Domingues K, Marmelo B, Reis L, Ruivo C, Guerreiro R, Lima R, Faria R, Marreiros A, Marques N. Short- and medium-term prognosis of Takotsubo syndrome in a Portuguese population. Rev Port Cardiol (Engl Ed). 2019;38(5):349-57. Epub 20190617. doi: 10.1016/j.repc.2018.07.010. PubMed PMID: 31221486.Mansencal N, El Mahmoud R, Pillière R, Dubourg O. Relationship between pattern of Tako-Tsubo cardiomyopathy and age: from midventricular to apical ballooning syndrome. Int J Cardiol. 2010;138(1):e18-20. Epub 20080803. doi: 10.1016/j.ijcard.2008.06.009. PubMed PMID: 18676042.
2. Del Buono MG, Montone RA, Camilli M, Gurgoglione FL, Ingrasciotta G, Meucci MC, Fracassi F, Niccoli G, Crea F. Takotsubo syndrome and left ventricular non-compaction cardiomyopathy: Casualty or causality? Auton Neurosci. 2019;218:64-7. Epub 20190228. doi: 10.1016/j.autneu.2019.02.008. PubMed PMID: 30890350.
3. Katsa I, Christia P, Massera D, Faillace R. Recurrent Stress Cardiomyopathy During COPD Exacerbation: Are Beta-adrenergic Agonists Only to Blame? Cureus. 2017;9(4):e1166. Epub 20170414. doi: 10.7759/cureus.1166. PubMed PMID: 28507838; PMCID: PMC5429155.
4. Mtour S, Abu-Hilal LH, Barghouthi DI, Njoum Y, Jabbarin F, Adwan B, Abu Asbeh I, Mtour A, Alsallamin I. Takotsubo cardiomyopathy prevalence and associated factors in patients presenting with a clinical picture of acute myocardial infarction in Palestine. Egypt Heart J. 2023;75(1):71. Epub 20230814. doi: 10.1186/s43044-023-00399-6. PubMed PMID: 37578674; PMCID: PMC10425303.
5. Nishida J, Kouzu H, Hashimoto A, Fujito T, Kawamukai M, Mochizuki A, Muranaka A, Kokubu N, Shimoshige S, Yuda S, Hase M, Tsuchihashi K, Miura T. "Ballooning" patterns in takotsubo cardiomyopathy reflect different clinical backgrounds and outcomes: a BOREAS-TCM study. Heart Vessels. 2015;30(6):789-97. Epub 20140725. doi: 10.1007/s00380-014-0548-x. PubMed PMID: 25059855.
6. Yan J, Madina M, Deng C, Yuan Q, Cao S, Xie X, Ma Y. Analysis of 9 Cases of Takotsubo Syndrome and an Analysis of the Clinical Characteristics of Takotsubo Syndrome From a Chinese Population. Front Cardiovasc Med. 2021;8:732193. Epub 20211026. doi: 10.3389/fcvm.2021.732193. PubMed PMID: 34765652; PMCID: PMC8576259.

**Wrong population (n=3)**

1. Réglat C, Chevalier JM, Coste P, Godon P, Renault L, Roudaut R, Seguy B, Gerbaud E. [Intermediate term outcome in 70 patients with Tako-Tsubo syndromes]. Ann Cardiol Angeiol (Paris). 2014;63(2):75-82. Epub 20140116. doi: 10.1016/j.ancard.2014.01.008. PubMed PMID: 24485824.
2. Rotondi F, Manganelli F. Recurrence of Tako-Tsubo syndrome, idiopathic dilated cardiomyopathy, and iterative ventricular tachycardia: just a fortuitous coincidence or a pathophysiological link? Eur Rev Med Pharmacol Sci. 2014;18(7):1111. PubMed PMID: 24763898.
3. Saito Y, Watanabe T, Ishigaki T, Toyoshima M, Katawaki W, Toshima T, Takahashi T, Yamanaka T, Watanabe M. Recurrent Takotsubo Syndrome Presenting with Different Ballooning Patterns and Electrocardiographic Abnormalities. Intern Med. 2023;62(20):2977-80. Epub 20230308. doi: 10.2169/internalmedicine.1564-23. PubMed PMID: 36889703; PMCID: PMC10641188.

**No outcomes of interest (n=6)**

1. Arcari L, Cacciotti L, Limite LR, Russo D, Sclafani M, Semeraro R, Ansalone G, Volpe M, Autore C, Musumeci MB. Clinical characteristics of patients with takotsubo syndrome recurrence: An observational study with long-term follow-up. Int J Cardiol. 2021;329:23-7. Epub 20201224. doi: 10.1016/j.ijcard.2020.12.047. PubMed PMID: 33359286.
2. Brenner R, Weilenmann D, Maeder MT, Jörg L, Bluzaite I, Rickli H, De Pasquale G, Ammann P. Clinical characteristics, sex hormones, and long-term follow-up in Swiss postmenopausal women presenting with Takotsubo cardiomyopathy. Clin Cardiol. 2012;35(6):340-7. Epub 20120409. doi: 10.1002/clc.21986. PubMed PMID: 22488168; PMCID: PMC6652298.
3. Looi JL, Wong CW, Khan A, Webster M, Kerr AJ. Clinical characteristics and outcome of apical ballooning syndrome in Auckland, New Zealand. Heart Lung Circ. 2012;21(3):143-9. Epub 20120110. doi: 10.1016/j.hlc.2011.11.010. PubMed PMID: 22237138.
4. Matteucci M, Ferrarese S, Corazzari C, Telli G, Mantovani V, Lorusso R, Beghi C. Ascending aortic aneurysm repair in the setting of Tako-tsubo cardiomyopathy. Perfusion. 2021;36(4):429-31. Epub 20200820. doi: 10.1177/0267659120950206. PubMed PMID: 32815793.
5. Opolski G, Pawlak MM, Roik MF, Kochanowski J, Scisło P, Piatkowski R, Kochman J, Karpiński G, Kowalik R, Grabowski M, Balsam P, Filipiak KJ. Clinical presentation, treatment, and long-term outcomes in patients with takotsubo cardiomyopathy. Experience of a single cardiology center. Pol Arch Med Wewn. 2010;120(6):231-6. PubMed PMID: 20567207.
6. Parodi G, Bellandi B, Del Pace S, Barchielli A, Zampini L, Velluzzi S, Carrabba N, Gensini GF, Antoniucci D. Natural history of tako-tsubo cardiomyopathy. Chest. 2011;139(4):887-92. Epub 20100930. doi: 10.1378/chest.10-1041. PubMed PMID: 20884730.

**Appendix 5:**

**Supplementary Table 4: Risk of bias assessment for included studies using the ROBINS-I risk of bias tool**

| **Study** | **Bias Due to Confounding** | **Bias in Selection of Participants** | **Bias in Classification of Interventions** | **Bias Due to Deviations from Intended Interventions** | **Bias Due to Missing Data** | **Bias in Measurement of Outcomes** | **Bias in Selection of the Reported Result** | **Overall Judgment** |
| --- | --- | --- | --- | --- | --- | --- | --- | --- |
| **Almendro-Delia et al. (2018)** | Moderate | Low to Moderate | Moderate | Low | Moderate | Low | Low | Moderate |
| **Cacciotti et al. (2012)** | Moderate | Low to Moderate | Moderate | Low | Moderate | Low | Low | Moderate |
| **Citro et al. (2019)** | Moderate | Low to Moderate | Low | Low | Moderate | Low | Low | Moderate |
| **D'Ascenzo et al. (2020)** | Moderate | Low to Moderate | Low | Low | Moderate | Low | Low | Moderate |
| **Elesber et al. (2007)** | Moderate | Low to Moderate | Low | Low | Moderate | Low | Low | Moderate |
| **Gopalakrishnan et al. (2015)** | Moderate | Low | Low | Low | Moderate | Low | Low | Moderate |
| **Kim et al. (2018)** | Moderate | Low | Low | Low | Moderate | Low | Low | Moderate |
| **Lau et al. (2021)** | Moderate | Low | Low | Low | Moderate | Low | Low | Moderate |
| **Novo et al. (2023)** | Moderate | Low | Low | Low | Moderate | Low | Low | Moderate |
| **Pereyra et al. (2021)** | Moderate | Low | Low | Low | Moderate | Low | Low | Moderate |
| **Raposeiras-Roubín et al. (2023)** | Moderate | Low | Low | Low | Moderate | Low | Low | Moderate |
| **Silverio et al. (2022)** | Moderate | Low | Low | Low | Moderate | Low | Low | Moderate |
| **Silverio et al. (2023)** | Moderate | Low to Moderate | Low to Moderate | Low | Moderate | Low | Low | Moderate |


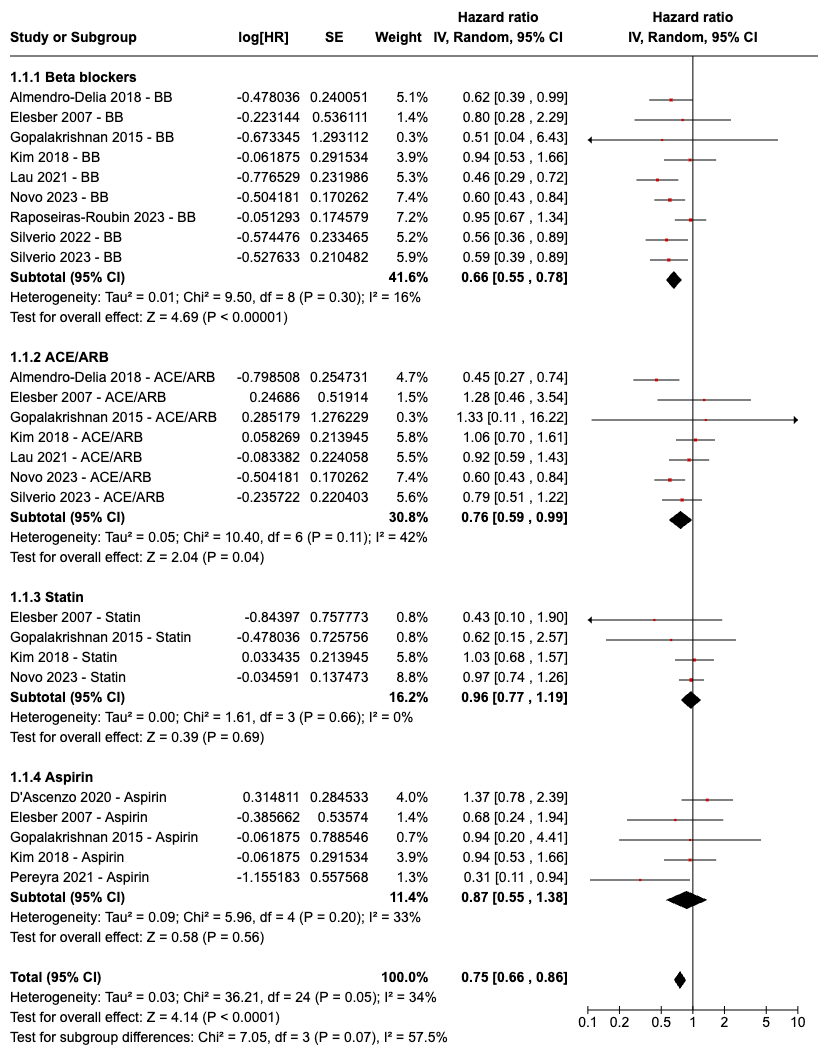


**Figure 4: Forest Plot Illustrating the Comparative Impact of Secondary Prevention Medications on All-Cause Mortality in Takotsubo Cardiomyopathy**
